# The complement of protein kinases of the microsporidium *Encephalitozoon cuniculi *in relation to those of *Saccharomyces cerevisiae *and *Schizosaccharomyces pombe*

**DOI:** 10.1186/1471-2164-8-309

**Published:** 2007-09-04

**Authors:** Diego Miranda-Saavedra, Michael JR Stark, Jeremy C Packer, Christian P Vivares, Christian Doerig, Geoffrey J Barton

**Affiliations:** 1 College of Life Sciences, University of Dundee, Dow St, Dundee DD1 5EH, Scotland, UK; 2 Division of Advanced Technologies, Abbott Laboratories, 100 Abbott Park Road, Abbott Park, IL 60064, USA; 3 Laboratoire de Parasitologie Moléculaire et Cellulaire. UMR CNRS 6023, Université Blaise Pascal, Aubière, France; 4 INSERM U609, Wellcome Centre for Molecular Parasitology, Glasgow Biomedical Research Centre, 120 University Place, Glasgow G12 8TA, Scotland, UK; 5 Cambridge Institute for Medical Research, Wellcome Trust/MRC Building, Addenbrooke's Hospital, Hills Road, Cambridge CB2 0XY, UK

## Abstract

**Background:**

Microsporidia, parasitic fungi-related eukaryotes infecting many cell types in a wide range of animals (including humans), represent a serious health threat in immunocompromised patients. The 2.9 Mb genome of the microsporidium *Encephalitozoon cuniculi *is the smallest known of any eukaryote. Eukaryotic protein kinases are a large superfamily of enzymes with crucial roles in most cellular processes, and therefore represent potential drug targets. We report here an exhaustive analysis of the *E. cuniculi *genomic database aimed at identifying and classifying all protein kinases of this organism with reference to the kinomes of two highly-divergent yeast species, *Saccharomyces cerevisiae *and *Schizosaccharomyces pombe*.

**Results:**

A database search with a multi-level protein kinase family hidden Markov model library led to the identification of 29 conventional protein kinase sequences in the *E. cuniculi *genome, as well as 3 genes encoding atypical protein kinases. The microsporidian kinome presents striking differences from those of other eukaryotes, and this minimal kinome underscores the importance of conserved protein kinases involved in essential cellular processes. ~30% of its kinases are predicted to regulate cell cycle progression while another ~28% have no identifiable homologues in model eukaryotes and are likely to reflect parasitic adaptations. *E. cuniculi *lacks MAP kinase cascades and almost all protein kinases that are involved in stress responses, ion homeostasis and nutrient signalling in the model fungi *S. cerevisiae *and *S. pombe*, including AMPactivated protein kinase (Snf1), previously thought to be ubiquitous in eukaryotes. A detailed database search and phylogenetic analysis of the kinomes of the two model fungi showed that the degree of homology between their kinomes of ~85% is much higher than that previously reported.

**Conclusion:**

The *E. cuniculi *kinome is by far the smallest eukaryotic kinome characterised to date. The difficulty in assigning clear homology relationships for nine out of the twentynine microsporidian conventional protein kinases despite its compact genome reflects the phylogenetic distance between microsporidia and other eukaryotes. Indeed, the *E. cuniculi *genome presents a high proportion of genes in which evolution has been accelerated by up to four-fold. There are no orthologues of the protein kinases that constitute MAP kinase pathways and many other protein kinases with roles in nutrient signalling are absent from the *E. cuniculi *kinome. However, orthologous kinases can nonetheless be identified that correspond to members of the yeast kinomes with roles in some of the most fundamental cellular processes. For example, *E. cuniculi *has clear orthologues of virtually all the major conserved protein kinases that regulate the core cell cycle machinery (Aurora, Polo, DDK, CDK and Chk1). A comprehensive comparison of the homology relationships between the budding and fission yeast kinomes indicates that, despite an estimated 800 million years of independent evolution, the two model fungi share ~85% of their protein kinases. This will facilitate the annotation of many of the as yet uncharacterised fission yeast kinases, and also those of novel fungal genomes.

## Background

The microsporidian *Encephalitozoon cuniculi *is a small spore-forming unicellular eukaryote leading an obligate intracellular parasitic lifestyle [1]. Inside a parasitophorous vacuole, the life cycle comprises three major phases: invasion with a polar tube system, proliferation with binary fission (merogony), and spore differentiation. Mitosis is of the closed type and dense structures called 'spindle pole bodies' resemble those of yeast. Chitin, a major polysaccharide of the fungal cell wall, is present in the inner part of the microsporidian spore wall. Trehalose, a disaccharide frequently found in fungi, has also been detected in microsporidia. The parasite's infections have medical importance since its hosts include various mammals, including humans, where it is known to cause digestive and clinical syndromes affecting the nervous system in HIV-infected or cyclosporine-treated patients [1].

The small and compact 2.9 Mb genome of *E. cuniculi *has recently been sequenced and characterised [2,3]. It split into 11 linear chromosomes harbouring 1,997 protein-coding sequences in a tightly clustered configuration. This degree of compaction has been achieved partly by reducing rDNA sequences as well as many protein-coding genes and intergenic regions [3]. *E. cuniculi *is therefore a microbial eukaryote that is highly-adapted to its parasitic lifestyle, and its genome sequence provides an opportunity for cataloguing the proteins that constitute its signal transduction networks. This understanding should shed light into the molecular mechanisms of pathogenicity and, from a wider perspective, on the minimal protein kinase-based signal transduction requirements of a eukaryotic intracellular parasite.

Reversible protein phosphorylation plays a central role in most cellular processes (in eukaryotic cells ~30% of proteins carry phosphate groups, [4,5]). Deregulation of protein phosphorylation is at the origin of several pathologies (e.g. cancers and neurodegenerative diseases) and protein kinases are now considered promising drug targets [see e.g. [6,7]]. Indeed, the first kinase inhibitors to be developed as drugs have recently been made available on the market [8,9].

The currently accepted classification of protein kinases splits the protein kinase superfamily into 'conventional' protein kinases (ePKs) and 'atypical' protein kinases (aPKs). ePKs are the largest group and have been sub-classified into 8 families by examining sequence similarity between catalytic domains, the presence of accessory domains, and by considering modes of regulation [10,11]. The 8 ePK families are: the AGC family (including cyclic-nucleotide and calciumphospholipiddependent kinases, ribosomal S6-phosphorylating kinases, Gprotein-coupled kinases, and all close relatives of these groups); the CAMKs (calmodulin-regulated kinases); the CK1 family (casein kinase 1, and close relatives); the CMGC family (including cyclin-dependent kinases, mitogen-activated protein kinases, glycogen synthase kinases [GSK3], and CDK-like kinases [CKLs]); the RGC family (receptor guanylate cyclase, which are similar in domain sequence to tyrosine kinases); the STE family (including many kinases functioning in MAP kinase cascades); the TK family (tyrosine kinases); and the TKL family (tyrosine kinase-like kinases). A ninth group, called the 'Other' group, consists of a mixed collection of kinases that could not be classified easily into the previous ePK families. The aPKs are a small set of protein kinases that do not share clear sequence similarity with ePKs but have been shown experimentally to have protein kinase activity [11], and comprise the following *bona fide *families [12]: Alpha (exemplified by myosin heavy chain kinase of *Dictyostelium discoideum*); PIKK (phosphatidyl inositol 3' kinase-related kinases); PDHK (pyruvate dehydrogenase kinases); and RIO ('*ri*ght *o*pen reading frame' as it was one of two adjacent genes that were found to be transcribed divergently from the same intergenic region [13]).

Protein kinases controlling the proliferation and development of parasitic eukaryotes represent attractive drug targets, because (i) they are likely to be essential to parasite multiplication and/or development; and (ii) these enzymes display structural and functional divergence when compared to their mammalian counterparts, suggesting that specific inhibition can be achieved [14-16]. Furthermore, the importance of protein kinases in most crucial cellular processes makes them interesting subjects of fundamental investigations into the cell biology of parasitic eukaryotes. The availability of the entire genome sequences of several parasites permits the study of their protein kinase complements (their 'kinomes'). Hence, two recent studies [17,18] reported the characterisation of the kinomes of the malaria parasite *Plasmodiumfalciparum*, showing that this organism possesses 85 (or 99, depending on the criteria used in the two studies) conventional protein kinases. A more recent and comparative study of the kinomes of all Apicomplexa species whose genome sequence is available has found the number of proteins kinases for the *P. falciparum *kinome to be 87ePKs and 3aPKs (Miranda-Saavedra, D. *et al*, manuscript submitted). The published kinomes of the Trypanosomatid species *Leishmaniamajor, Trypanosoma brucei *and *Trypanosoma cruzi *indicate that these parasites harbour between 176 (*T. brucei*) and 199 (*L. major*) kinases, most of which are orthologous across the three Trypanosomatid species [19]. These kinomes compare to kinomes of 478, 115 and 106 conventional protein kinases in human, fission yeast and budding yeast, respectively [11,20,21]. Here, we present an analysis of the kinome of *E. cuniculi*, and show that this organism has the smallest characterised kinome of all eukaryotes examined to date.

## Results and discussion

### The kinome of *E. cuniculi*

The 1,997 predicted peptides of *E. cuniculi *were scanned with a multi-level HMM library of the kinase catalytic domain. This library is especially sensitive for retrieving kinase catalytic domain sequences from databases and, at the same time, does an automatic classification of kinases into families [12]. The application of the HMM library retrieved 32 protein kinases in *E. cuniculi *(Table 1), two of which lacked critical residues that confer catalytic activity and which may therefore be pseudokinases [22]. The HMM library has also been shown to be selective enough to classify some kinases of the 'Other' group of *S. cerevisiae *into the main ePK families [12]. Among the ePKs, *E. cuniculi *was found to harbour 4 kinases of the AGC family, 5 CAMKs, 2 CK1s, 12 CMGCs, 1 TKL, and 5 kinases which, by complete clustering analysis with the kinomes of *S. cerevisiae *and *S. pombe *(see below), were found to belong to the 'Other' group. *E. cuniculi *was also found to encode 3 atypical protein kinases (aPKs): 2 of the PIKK family and one of the RIO family. No kinases of the ePK families RGC, TK, or FIKK (a family identified in *P. falciparum *and apparently specific to Apicomplexa), or of the aPK families Alpha or PDHK, were found.

**Table 1:** The 32 protein kinases of *E. cuniculi *split by kinase family

**Kinase family**	**Protein Id**	**Size (amino acids)**	**UniProt**	**Predicted Catalytic Activity**
**AGC**	CAD25005.1	871	Q8SSJ0	Active
	CAD25568.1	272	Q8SRL5	Active
	CAD25584.1	322	Q8SRK8	Active
	CAD25776.1	348	Q8SR57	Inactive (K, D^1^)
**CAMK**	CAD26060.1	300	Q8SQT4	Active
	CAD25086.1	516	Q8SSH1	Active
	CAD26208.1	414	Q8SSA8	Active
	CAD26452.1	467	Q8SR98	Active
	CAD26242.1	566	Q8SW31	Active
**CK1**	CAD26235.1	327	Q8SS96	Active
	CAD26108.1	318	Q8SQR2	Active
**CMGC**	CAD25082.1	586	Q8SSH4	Active
	CAD26483.1	309	Q8SR90	Active
	CAD25174.1	308	Q8SW92	Active
	CAD26671.1	319	Q8SRU0	Active
	CAD25660.1	350	Q8SRI3	Active
	CAD26328.1	265	Q8SRF5	Active
	CAD26006.1	329	Q8SQW2	Active
	CAD26495.1	296	Q8SR86	Active
	CAD26039.1	328	Q8SQU8	Active
	CAD26498.1	351	Q8SR85	Active
	CAD25731.1	351	Q8SR83	Active
	CAD25928.1	405	Q8SQZ4	Active
**TKL**	CAD26582.1	694	Q8SRY2	Active
**Other**	CAD26233.1	454	Q8SW35	Active
	CAD26466.1	363	Q8SR94	Active
	CAD24933.1	488	Q8SWM6	Active
	CAD26209.1	390	Q8SSA7	Inactive (K)
	CAD25400.1	185	Q8SVD9	Active
**PIKK**	CAD25142.1	1935	Q8SSE7	
	CAD25955.1	1433	Q8SQY7	
**RIO**	CAD26627.1	409	Q8SV17	

The largest protein kinase family in *E. cuniculi *was thus found to be the CMGC, most members of which are involved in the control of cell proliferation. The CMGC is also the largest family in Trypanosomatids [19], in *P. falciparum *[17], and in other Apicomplexa (Miranda-Saavedra, D. *et al*., submitted). Interestingly, no kinases of the STE or NIMA families were found, explaining the lack of success in amplifying microsporidian MAPK homologues by PCR (Thellier and Doerig, unpublished). It has been noted that *P. falciparum *also lacks canonical 3-component MAPKKK-MAKK-MAPK cascades [17]. The three Trypanosomatid species *L. major, T. brucei *and *T. cruzi *are known to possess kinases of the Ste7, Ste11, and Ste20 subfamilies [19]. Their kinomes are ~2.5 times larger than that of *P. falciparum*, suggesting that MAP kinase cascades might have an ancestral origin, and which might have been lost in streamlined kinomes such as those of *E. cuniculi *or the malaria parasite.

TKLs have previously been found in Metazoa, Apicomplexan parasites, *Entamoeba histolytica*, and the plants *Arabidopsis thaliana *and *Oryza sativa*. These findings, together with the observation that the fungi *Cryptococcus neoformans *and *Phanerochaetechrysosporium *contain putative TKLs, suggests that TKLs have been lost secondarily from most fungal lineages [12]. Therefore, the finding of a putative TKL in *E. cuniculi *is not too surprising. The sole member of the TKL family in the microsporidian kinome represents the only instance of a protein kinase family found in *E. cuniculi *that is not represented in either *S. cerevisiae *or *S. pombe *(Table 1).

### The kinomes of *S. cerevisiae *and *S. pombe *and their homology relationships with the *E. cuniculi *kinome

The kinome of *S. cerevisiae *was the first to be described [21], followed by those of several higher eukaryotic organisms published by the same group and now available through KinBase [23]. The budding yeast kinome consists of 115 conventional protein kinase (ePK) and 9 atypical protein kinase (aPK) sequences. It has previously been noted that among the aPKs, there is evidence for protein phosphorylation activity only for members of the PIKK, PDHK, RIO, and Alpha families [12]. The kinome of *S. pombe *was described recently [20] as part of a study that incorporated the systematic deletion of each of the fission yeast kinases and analysis of the mutant phenotype. Bimbó *et al*. [20] identified 106 ePKs by interrogating public databases for sequences that had been annotated as kinases, but the aPKs were not considered. Computational analysis suggested that fission yeast contains no tyrosine kinases [20]. Thirty-one ePKs were considered as likely to have a 'tyrosine kinase signature' (although the identity of this signature was not indicated) and 67/106 (63.2%) ePKs were found to have direct homologues in *S. cerevisiae *as defined by mutual best-hit analysis. Deletion analysis of ePK genes indicated that 17/106 ePKs (16%) are essential for cell viability. Of the remaining 89 ePKs, deletion phenotypes were assessed under various stress conditions. 46% of these non-essential ePKs of fission yeast were found to exhibit hypersensitivity to at least one of the 17 stress factors tested, allowing the functional grouping of fission yeast ePKs into 4 major signalling pathways according to the nature of the stress.

We have carried out an independent database search for ePKs and aPKs in *S. pombe *and have identified 3 additional ePKs, one of which is the fission yeast homologue of *S. cerevisiae *Bud32p (SPAP27G11.07: Table 2). Thus, the kinome of *S. pombe *was found to consist of 109 ePKs and 8 aPKs (Table 2). Both the kinomes of *S. cerevisiae *and *S. pombe *lack kinases of the families RGC, TK, and Alpha. Phylogenetic analysis suggests that 91/109 (83.5%) of ePKs of fission yeast share a homologue in *S. cerevisiae*; likewise 96/115 (83.5%) of ePKs of *S. cerevisiae *have a homologue in *S. pombe*. With the inclusion of aPKs (i.e. considering the two complete kinomes) 100/117 (85.5%) of *S. pombe *kinases have homologues in *S. cerevisiae *(Table 3). The same was found to be true for 104/124 (83.9%) of *S. cerevisiae *kinases. Therefore, the degree of homology between the kinomes of the two fungi is ~20% greater than previously reported [20]. Part of the improvement is because the multilevel HMM library produces an automatic classification of protein kinases into families. The advantage of doing family-family comparisons, and the subsequent generation of multiple alignments and phylogenetic analysis for each family, is that proteins that are closer sequence-wise make better alignments. Since a phylogenetic tree is only as good as the underlying alignment, splitting the kinases into families prior to generating phylogenies is a more powerful method for comparing entire kinomes. Since our analysis shows that there are up to 16 instances of potentially redundant paralogous ePK pairs in fission yeast (Table 3) it is likely that the proportion that are essential has been underestimated if paralogue pairs that can complement each other's function are included, and the real value is probably over 20%. Similar considerations in budding yeast also suggest that over 20% of protein kinase activities are essential for vegetative growth.

**Table 2:** The protein kinase complements of *E. cuniculi, S. cerevisiae *and *S. pombe*, split by family

	**ePKs**							
	
	**AGC**	**CAMK**	**CK1**	**CMGC**	**STE**	**TKL**	**Other**	**Total**
**EC**	4	5	2	12	0	1	5	29
**SC**	20	37	4	25	14	0	15	115
**SP**	20	28	5	26	13	0	17	109

	**aPKs**							
				
	**PIKK**	**RIO**	**PDHK**	**Alpha**	**Total**			
			
**EC**	2	1	0	0	3			
**SC**	5	2	2	0	9			
**SP**	5	2	1	0	8			

**Table 3:** Homology relationships between the kinomes of *S. cerevisiae, S. pombe *and *E. cuniculi*

**AGC family**				
				
**Sub-family**	***S. cerevisiae***	***S. pombe***	***E. cuniculi***	**Deletion mutant**
AKT	*YPK1, YPK2*	SPCC24B10.07 (*gad8*)		Viable (Sc, Sp)
AKT	*SCH9*	SPAC1B9.02c (*sck1*), SPAC22E12.14c (*sck2*)		Viable (Sc, Sp)
AUR	*IPL1*	SPCC320.13c (*ark1*)	CAD25568.1	Lethal (Sc, Sp)
BUB	*BUB1*	SPCC1322.12c (*bub1*)		Viable (Sc, Sp)
NDR	*RIM15*	SPAPB18E9.02c (*ppk18*), SPCC1450.11c (*cek1*)		Viable (Sc, Sp)
NDR	*CBK1*	SPAC821.12 (*orb6*)	CAD25005.1	Lethal (Sc, Sp)
NDR	*DBF2, DBF20*	SPCC417.06c (*ppk35*), SPAC24B11.11c (*sid2*)		Lethal (*sid2*)
PDK1	*PKH1, PKH2*	SPCC576.15c (*ksg1*)		Lethal (*ksg1*)
PDK1	*YDR466W (PKH3)*	SPBC1778.10c (*ppk21*)		Viable (Sc, Sp)
PKA	*TPK1, TPK2, TPK3*	SPBC106.10 (*pka1*)	CAD25584.1	Viable (Sc, Sp)
PKC	*PKC1*	SPAC17G8.14c (*pck1*), SPBC12D12.04c (*pck2*)		Lethal (*PKC1*)
RSK	*KIN82*	SPBC1861.09 (*ppk22*)		Viable (Sc, Sp)
RSK	*YNR047W*	SPAC4G8.05 (*ppk14*)		Viable (Sc, Sp)
RSK	*YBR028C*	SPCC4G3.08 (*psk1*)		Viable (Sc, Sp)
	**AGC unpaired**	**AGC unpaired**	**AGC unpaired**	
	*YKL171W*	SPBC725.06c (*ppk31*)	CAD25776.1	
		SPCC162.10 (*cmk2*)		
				

**CAMK family**				
				
**Sub-family**	***S. cerevisiae***	***S. pombe***	***E. cuniculi***	**Deletion mutant**

CAMK1	*CMK1, CMK2*	SPACUNK12.02c (*cmk1*)		Viable (Sc, Sp)
CAMK1	*RCK1, RCK2*	SPAC23A1.06c (*cmk2*), SPCC1322.08 (*srk1*)		Viable (Sc, Sp)
CAMKK	*TOS3, PAK1*	SPCC297.03 (*ssp1*), SPCC1919.01 *(ppk34*)		Viable (Sc, Sp)
CAMKL	*CHK1*	SPCC1259.13 (*chk1*)	CAD26208.1	Viable (Sc, Sp)
CAMKL	*KIN1, KIN2*	SPBC4F6.06 (*kin1*), SPBC32C12.03c (*ppk25*)	CAD26242.1, CAD26452.1	Viable (Sc, Sp)
CAMKL	*KIN4, YPL141C*	SPAC110.01 (*ppk1*)		Viable (Sc, Sp)
CAMKL	*YPL150W*	SPAC890.03 (*ppk16*)		Viable (Sc, Sp)
CAMKL	*FUN31, YOL045W*	SPAC1805.01c (*ppk6*)		Viable (Sc, Sp)
CAMKL	*SNF1*	SPCC74.03c (*ssp2*), SPAC23H4.02 (*ppk9*)		Viable (Sc, Sp)
CAMKL	*KCC4, GIN4, HSL1*	SPAC644.06c (*cdr1*), SPAC57A10.02 (*cdr2*)		Viable (Sc, Sp)
CAMK-Unique	*MEK1*	SPAC14C4.03 (*mek1*)		Viable (Sc, Sp)
CAMK-Unique	*PRR1*	SPBC337.04 (*ppk27*)		Viable (Sc, Sp)
HAL	*SAT4*	SPAC29A4.16 (*ppk10*)		Viable (Sc, Sp)
HAL	*YOR267C, YDL025C*	SPCC1020.10 (*oca2)*		Viable (Sc, Sp)
RAD53	*RAD53*	SPCC18B5.11c (*cds1*)		Lethal (*RAD53*)
RAN	*KSP1*	SPBC16E9.13 (*ppk20*)		Viable (Sc, Sp)
ULK	*APG1*	SPCC63.08c (*ppk36*)		Viable (Sc, Sp)
	**CAMK unpaired**	**CAMK unpaired**	**CAMK unpaired**	
	*(PTK1, PTK2)*	SPAC22G7.08 (*ppk8*)	CAD26060.1	
	*(HAL5, KKQ8)*	(SPCC70.05c (*ppk37*, Lethal), SPBC21.07c (*ppk24*))	CAD25086.1	
	*DUN1*	*SPBC19C2.05*		
	*YMR291W*	*SPAC15A10.13*		
	*(NPR1, PRR2)*	*SPBP23A10.10*		
	*IKS1*			
	*YGR052W*			
	*ISR1*			
				

**CK1 family**				
				
**Sub-family**	***S. cerevisiae***	***S. pombe***	***E. cuniculi***	**Deletion mutant**

CK1	*YCK1*	SPAC1805.05 (*cki3*)		
CK1	*YCK2*	SPBC1347.06c (*cki1*)		
CK1	*YCK3*	SPBP35G2.05c (*cki2*)		
CK1	*HRR25*	SPBC3H7.15 (*hhp1*)	CAD26235.1	Lethal (*HRR25*)
		SPAC23C4.12 (*hhp2*)	CAD26108.1	
				

**CMGC family**				
				
**Sub-family**	***S. cerevisiae***	***S. pombe***	***E. cuniculi***	**Deletion mutant**

CDC7	*CDC7*	SPBC776.12c (*hsk1*), SPBC21C3.18 (*spo4*)	CAD26498.1, CAD25731.1	Lethal (*CDC7*, *hsk1*)
CDK	*KIN28*	SPBC19F8.07 (*crk1*)	CAD25174.1	Lethal (*KIN28*, *crk1*)
CDK	*CDC28*	SPBC11B10.09 (*cdc2*)	CAD26495.1	Lethal (*CDC28*, *cdc2*)
CDK	*CTK1*	SPAC2F3.15 *(lsk1*)	CAD26006.1	Viable (Sc, Sp)
CDK	*CAK1*	SPAC1D4.06c (*csk1*)		Lethal (*CAK1*)
CDK	*SGV1*	SPBC32H8.10 (*cdk9*)		Lethal (*SGV1*, *cdk9*)
CDK	*PHO85*	SPCC16C4.11 (*pef1*)		Viable (Sc, Sp)
CDK	*SSN3*	SPAC23H4.17c (*srb10*)		Viable (Sc, Sp)
CK2	*CKA1, CKA2*	SPAC23C11.11 (*cka1*)	CAD26671.1	Lethal (*cka1*)
CLK	*KNS1*	SPAC1D4.11c (*lkh1*)		Viable (Sc, Sp)
DYRK	*YAK1*	SPAC823.03 (*ppk15*)	CAD25928.1	Viable (Sc, Sp)
GSK	*RIM11, MRK1*	SPBC8D2.01 (*gsk31*), SPAC1687.15 (*gsk3*)		Viable (Sc, Sp)
MAPK	*FUS3, KSS1*	SPAC31G5.09c (*spk1*)		Viable (Sc, Sp)
MAPK	*HOG1*	SPAC24B11.06c (*sty1*)		Viable (Sc, Sp)
MAPK	*SLT2, YKL161C*	SPBC119.08 (*pmk1*)		Viable (Sc, Sp)
RCK	*IME2*	SPAC3C7.06c (*pit1*), SPBC8D2.19 (*mde3*)		Viable (Sc, Sp)
SRPK	*SKY1*	SPBC530.14c (*dsk1*)		Viable (Sc, Sp)
TTK	*MPS1*	SPBC106.01 (*mph1*)	CAD25082.1	Lethal (*MPS1*)
	**CMGC unpaired**	**CMGC unpaired**	**CMGC unpaired**	
	*SMK1*	SPAC16C9.07 (*ppk5*), SPAC2F7.03c (*pom1*)	CAD25660.1	
	*(MCK1, YOL128C)*	SPCC777.14 (*prp4*, Lethal)	(CAD26039.1, CAD26483.1)	
		*SPBC336.14C*		
		SPBC18H10.15 (*ppk23*)	CAD26328.1	
				

**STE family**				
				
**Sub-family**	***S. cerevisiae***	***S. pombe***	***E. cuniculi***	**Deletion mutant**

STE7	*PBS2*	SPBC409.07c (*wis1*)	(none)	Viable (Sc, Sp)
STE7	*MKK1, MKK2*	SPBC543.07 (*pek1*)		Viable (Sc, Sp)
STE7	*STE7*	SPAC1D4.13 (*byr1*)		Viable (Sc, Sp)
STE20	*STE20*	SPBC1604.14c (*shk1*)		Lethal (*shk1*)
STE20	*CLA4, SKM1*	SPAC1F5.09c (*shk2*)		Viable (Sc, Sp)
STE20	*SPS1*	SPAC2C4.14c (*ppk11*), SPAC9G1.09 (*sid1*)		Lethal (*sid1*)
STE20	*CDC15*	SPBC21.06c (*cdc7*)		Lethal (*CDC15*, *cdc7*)
STE20	*KIC1*	SPBC17F3.02 *(nak1*)		Lethal (*KIC1*, *nak1*)
STE11	*SSK2, SSK22*	SPAC1006.09 (*win1*), SPAC9G1.02 (*wis4*)		Viable (Sc, Sp)
STE11	*STE11*	SPBC1D7.05 (*byr2*)		Viable (Sc, Sp)
STE11	*BCK1*	SPAC1F3.02c (*mkh1*)		Viable (Sc, Sp)
				

**Other group**				
				
**Sub-family**	***S. cerevisiae***	***S. pombe***	***E. cuniculi***	**Deletion mutant**

Bud32	*BUD32*	*SPAP27G11.07c*^$$^	CAD25400.1	Viable (*BUD32*)
GCN2	*GCN2*	SPAC20G4.03c *(hri1*)		Viable (Sc, Sp)
IRE	*IRE1*	SPAC167.01 (*ppk4*)		Viable (Sc, Sp)
NAK	*YPL236C*	SPAC3H1.13 (*ppk13*)		Viable (Sc, Sp)
NAK	*AKL1, PRK1, ARK1*	SPCP1E11.02 (*ppk38*), SPBC6B1.02 (*ppk30*), SPBC557.04 *(ppk29*)		Viable (Sc, Sp)
NEK	*KIN3*	SPAC19E9.02 (*fin1*)		Viable (Sc, Sp)
PLK	*CDC5*	SPAC23C11.16 (*plo1*)	CAD24933.1	Lethal (*CDC5*, *plo1*)
VPS15	*VPS15*	SPBC119.07 (*ppk19*)		Viable (Sc, Sp)
WEE	*SWE1*	SPCC18B5.03 (*wee1*), SPBC660.14 (*mik1*)	CAD26466.1	Viable (Sc, Sp)
	**Other unpaired**	**Other unpaired**	**Other unpaired**	
	*SCY1*	*SPBC15C4.02*^$$^	CAD26209.1	
	*ELM1*	SPAC12B10.14c (*ppk2*)	CAD26233.1	
		*SPAC23C4.03*^$$^		
		SPAC222.07c (*hri2*)		
		SPBC36B7.09 (*ppk28*)		
				

**TKL family**	***S. cerevisiae***	***S. pombe***	***E. cuniculi***	**Deletion mutant**

	(none)	(none)	CAD26582.1	Unknown
				

**PIKK family**	***S. cerevisiae***	***S. pombe***	***E. cuniculi***	**Deletion mutant**

	*MEC1*	*SPBC216.05*		
	*TEL1*	SPCC23B6.03c *	CAD25142.1, CAD25955.1	
	*TOR1*	*SPBC216.07c*		
	*TOR2*	*SPBC30D10.10c*		
	*TRA1*	*SPAC1F5.11c*		
				

**RIO family**	***S. cerevisiae***	***S. pombe***	***E. cuniculi***	

	*RIO1*	*SPAC10F6.10*		
	*RIO2*	*SPBC1703.05*	CAD26627.1	
				

**PDHK family**	***S. cerevisiae***	***S. pombe***	***E. cuniculi***	

	*YGL059W*		(none)	
	*YIL042C*	*SPAC644.11c*		

^$$^novel; no information on these *S pombe *kinases

* SPAC22E12.16c, SPBC577.06c, and SPAC458.05 are TEL1-related

The establishment of homology relationships between the kinomes of *S. cerevisiae *and *S. pombe *(Table 3), together with additional information extracted from the *Saccharomyces *Genome Database [24,25] and Pombase [26], sets a powerful scene for cross-annotating the kinomes of these model organisms and, by extension, of other fungal kinomes that will be characterised in the near future (Table 3). Homology relationships between the kinomes of *S. cerevisiae, S. pombe *and *E. cuniculi *will be discussed below on a family-by-family basis.

### AGC family

**Figure 1:**
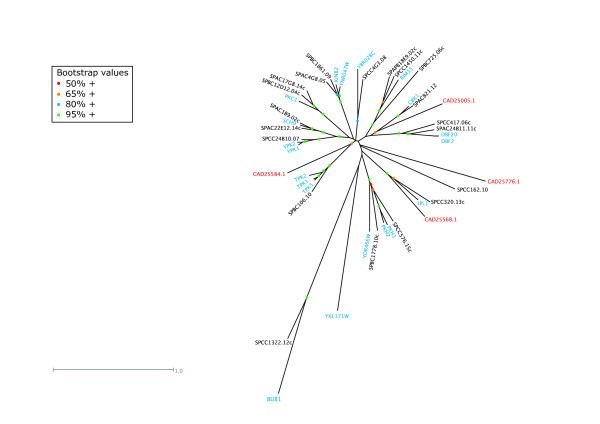
Unrooted tree of the AGC kinases of *S. cerevisiae *(blue), *S. pombe *(black) and *E. cuniculi *(red).

*E. cuniculi *was found to harbour four AGC kinases (a family which includes cyclic-nucleotide and calcium-phospholipid-dependent kinases, ribosomal S6-phosphorylating kinases, G protein-coupled kinases, and all close relatives of these groups), three of which have orthologues in the yeasts (Table 3, Fig. 1, Additional file 1). The previously characterised CAD25584.1 [27] is the microsporidian homologue of yeast PKA (encoded by *S. cerevisiae TPK1-3*) while CAD25568.1 is the homologue of the yeast Ipl1p/Ark1 Aurora kinases. PKA is universally found in eukaryotic cells and in both *S. cerevisiae *and *S. pombe *it is involved in nutrient sensing and signalling, sporulation and cellular stress responses [28-30]. Although this putative microsporidian PKA (CAD25584.1) is somewhat diverged from its yeast orthologues (Fig. 1), the identification of two PKA regulatory subunit homologues (CAD24891.1 and CAD25013.1) is consistent with the presence of PKA in *E. cuniculi *[27]. PKA has an essential function in budding yeast for vegetative growth [31] and is required in fission yeast for spore germination in response to glucose, but not for vegetative growth itself [20,28]. A major role of the *S. cerevisiae *Aurora kinase Ipl1p is to drive chromosome biorientation on the mitotic spindle by promoting detachment of kinetochores from microtubules when both sister chromatids are attached to microtubules from the same spindle pole [32,33]. The presence of an orthologue in *E. cuniculi *(CAD25568.1) and its binding partner Sli15 (CAD26983.1) suggests that this vital role is likely to be conserved in all eukaryotes, although Ipl1p, Ark1 and their mammalian homologues have additional cell cycle roles to play [34-37] that may also be important in the microsporidian.

Of the two remaining orphan *E. cuniculi *kinases in this group, one is predicted to be inactive (CAD25776.1; Table 1) while the second (CAD25005.1) clusters with yeast Cbk1p/Orb6 and Dbf2p/Dbf20p/Sid2 (Fig. 1). These yeast kinases are all essential (*DBF2 *and *DBF20 *are redundant and the gene knockouts show synthetic lethality in budding yeast:[38]) and share with CAD25005.1 a protein kinase C terminal domain (PF00433) that follows the protein kinase domain itself. Dbf2p and Dbf20p function in the budding yeast mitotic exit network (MEN) while fission yeast Sid2 is part of the analogous septation initiation network (SIN) in fission yeast [39,40]. However, since the Cdc15p/Cdc7 kinases that also function as essential components of these pathways are not present in *E. cuniculi *(see below), it is more likely that CAD25005.1 is functionally related to Cbk1p/Orb6. If *E. cuniculi *does lack a MEN/SIN pathway this may reflect a reduced need to coordinate cytokinesis with nuclear division, perhaps because the relative timing of these events is sufficient to ensure high fidelity division without specific mechanisms to coordinate them. In budding yeast the MEN is also critical for promoting inactivation of mitotic cyclin-dependent kinase activity (CDK) through release of the Cdc14p phosphatase from the nucleolus, but this role is not conserved in fission yeast [41] and so may also not be vital in *E. cuniculi*. In budding yeast, Cbk1 is required for regulating cellular morphogenesis and the expression of genes involved in cell separation [42] and in fission yeast it may have an analogous role in coordinating morphogenesis and cell division [43]. These roles may therefore be highly conserved if CAD25005.1 is genuinely functionally related to these yeast kinases. However, CAD25005.1 also has a phorbol ester/diacylglycerol-binding C1 domain (PF00130) that is shared with yeast protein kinase Cs, although they are located at the opposite end of the polypeptide to their position in, for example, budding yeast Pkc1p, and there is no C2 domain (PF00168). Thus perhaps the most likely scenario is that CAD25005.1 represents a somewhat divergent PKC that, like its yeast counterparts, may be activated by Rho GTPases [44-47]. In budding yeast, Pkc1p is involved in promoting cell wall integrity [see [48]], an essential role that it shares with its fission yeast homologues [45].

Regarding the remaining AGC kinases present only in the two yeasts, almost all show direct homology relationships except for budding yeast YKL171W and fission yeast's *ppk31 *and *ppk33*, which appear to be lineage-specific (Table 3). Where the function of the conserved kinases is known, it frequently concerns nutrient signalling or cell integrity, functions that may be less important for an obligate intracellular parasite such as *E. cuniculi*. The PDK1 homologues are essential in both yeasts [20,49,50], although the AKT homologues that should function downstream of the PDK1 homologues are essential only for vegetative growth in budding yeast [20]. Finally, since *E. cuniculi *lacks a homologue of the spindle checkpoint kinases Bub1p/Bub1 it is likely that this checkpoint mechanism is absent from the microsporidian. Although the spindle checkpoint is important in higher eukaryotes because of its role in the timing of mitotic events [see e.g. [51,52]], it is non-essential in yeast under normal circumstances and is apparently dispensable in *E. cuniculi*.

### CAMK family

**Figure 2:**
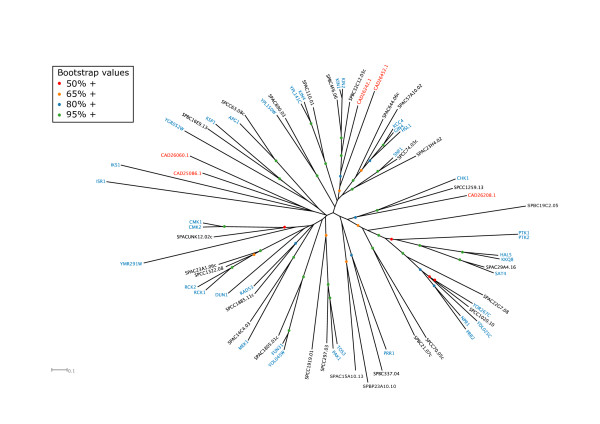
Unrooted tree of the CAMK kinases of *S. cerevisiae *(blue), *S. pombe *(black) and *E. cuniculi *(red).

Of the five CAMKs (calmodulin-regulated kinases) of *E. cuniculi*, only three could be shown to be homologues of yeast CAMKs, while the two other microsporidian CAMKs did not cluster with characterised fungal kinases within the same family ('semi-orphan'). CAD26208.1 is related to Chk1p/Chk1, while CAD26242.1 and CAD26452.1 are both related to Kin1p/Kin2p of budding yeast and Kin1/Ppk25 of fission yeast (Table 3, Fig. 2, Additional file 2). Given that budding yeast cells lacking both *KIN1 *and *KIN2 *are viable [53], finding two homologues of these kinases in *E. cuniculi *is surprising. In fission yeast, loss of just one of the two isoforms (Kin1p) produced a significant morphological defect [54], so perhaps simultaneous deletion of both isoforms will reveal a more critical role for this group of kinases in fission yeast. It also remains possible that a third budding yeast CAMK may function redundantly with Kin1p and Kin2p. Budding yeast Kin1p and Kin2p are the homologues of *C. elegans *Par-1 [55], a protein kinase essential for the establishment of anterior-posterior polarity in the onecell embryo and generally involved in the intracellular organisation cues in various biological systems. In *E. cuniculi*, the symmetric differentiation of the spore exhibits an evident anterio-posterior polarity and the Par-1 orthologue may have a role to play in this process. In fission yeast, loss of Kin1 causes monopolar growth because cells fail to activate polarized growth at their new end (termed NETO for new end take-off: [55]). In *S. cerevisiae*, polarised growth takes the form of asymmetric growth of the bud and localized fusion of secretory vesicles at the bud tip in G1 cells until later in the cell cycle, when re-polarisation to the bud neck occurs. This is regulated both by changes in the actin cytoskeleton and in the distribution of the secretory landmark protein Sec3p, a component of the Exocyst complex [56]. It is therefore interesting that Kin1p and Kin2p interact with multiple components of the exocytic machinery and may regulate the fusion of secretory vesicles with the cell surface [57]. Thus taken together, these considerations point to a potential role of the *E. cuniculi *homologues in polarized secretion.

The *E. cuniculi *CAMK CAD26208.1 is the orthologue of Chk1 kinase in the yeasts (Table 3, Fig. 2). Although nonessential in both yeast species, this kinase nonetheless plays a central role in the DNA damage response, delaying mitosis to allow time for DNA repair to occur and stimulating the expression of DNA damage repair functions [58]. In fact, in evolutionary terms Chk1 is considered the most ancient of the major cell cycle control kinases [59], which functions in a pathway downstream of a PIKK family member that is part of the damage sensing machinery [58]. The presence of a Mec1p/Tel1p-related PIKK kinase in *E. cuniculi *is therefore consistent with the presence of a Chk1 orthologue. Rad53p and Cds1 (orthologues of human Chk2 kinase) are also implicated in this checkpoint pathway, and are involved in stabilising stalled replication origins [60,61]. However, the relative importance of the Chk1 and Rad53p/Cds1/Chk2 arms for the response to DNA damage and stalled replication forks differs in different systems. Whereas Cds1 and Rad53 are dispensable in fission yeast, Rad53p and Mec1p are essential in budding yeast because of an additional role in regulating dNTP levels [62,63]. Thus the single Chk1-related kinase in *E. cuniculi *may play roles in both the replication and repair aspects of this pathway.

Perhaps the biggest surprise within this family of kinases is the apparent absence of an AMPK homologue (Snf1p in budding yeast), an enzyme previously thought to be ubiquitous in eukaryotes. All Apicomplexan genomes (*Plasmodium ssp. falciparum, berghei, chabaudi *and *yoelii, Cryptosporidium ssp. hominis *and *parvum, Theileria ssp. annulata *and *parva*, and *Toxoplasma gondii*) contain one AMPK orthologue (Miranda-Saavedra, D., *et al*., manuscript submitted), and *E. cuniculi *is therefore the first eukaryote to be described that lacks AMPK. AMPK has been termed the "fuel gauge of the cell", responding to the AMP/ATP ratio and downregulating ATPconsuming processes and upregulating ATP-generating processes in response to changing cellular energy balance [64]. In budding yeast, Snf1p has not been formally shown to be AMP regulated, but is critical for derepression of genes required for growth on non-fermentable carbon sources once glucose (and hence fermentative generation of ATP) has been exhausted [65]. In contrast, nothing is known about AMPK in fission yeast although it has two apparent orthologues. Consistent with the lack of an identifiable AMPK, *E. cuniculi *also lacks homologues of the protein kinases that are required to activate Snf1p (Tos3p, Pak1p and Elm1p, Table 3, [66]). Given that *E. cuniculi *has a limited ability to generate its own ATP and that it recruits host mitochondria close to its plasma membrane, it is likely that it imports host ATP using the four distinct ATP/ADP translocases that it encodes [2,67]. It may therefore have little capacity for controlling its energy balance except through ATP/ADP exchange with the host cell, and therefore effectively rely on regulation of host cell metabolism by the host cell's AMPK to ensure its own energy supply.

Budding yeast and fission yeast possess 11 and 6 lineage-specific CAMKs (i.e. with no identifiable orthologues in the other species: Table 3). The CAMKs not represented in *E. cuniculi *include budding yeast's Psk1p and Psk2p (FUN31 and YOL045W) (involved in nutrient sensing and metabolic regulation: [66]), Hrk1p (YOR267C) and Ptk2p (involved in plasma membrane ATPase regulation: [68]); Kcc4p/Gin4p/Hsl1p/Cdr1/Cdr2 (cell cycle regulators through phosphorylation of Swe1p/Wee1: [69,70]).

### CK1 family

**Figure 3:**
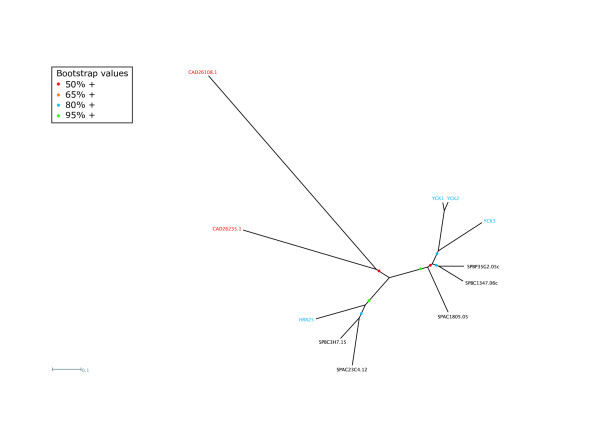
Unrooted tree of the CK1 kinases of *S. cerevisiae *(blue), *S. pombe *(black) and *E. cuniculi *(red).

The two microsporidian CK1s (casein kinase 1 and close relatives) cluster with the fungal homologues of Hrr25p (Table 3, Fig. 3, Additional file 3), and lack the Cterminal palmitoylation signal required for plasma membrane localization found in the three budding yeast Yck CK1 kinases [71,72]. Budding yeast Hrr25p is essential, as is the presence of at least one of the two redundant Yck1p and Yck2p isoforms [73]. Although the *S. pombe *Hhp1 and Hhp2 appear to be co-evolution paralogues of yeast Hhr25p, cells lacking both genes are viable [74]. CK1s in general and *S. cerevisiae's *Hrr25 in particular have been ascribed a wide range of functions [75] including vesicular trafficking, regulation of gene expression and DNA repair in yeast. One critical role in both yeast species is as part of a mechanism for ensuring monopolar attachment of sister kinetochores in meiosis I, a phenomenon that is essential for ensuring correct disjunction of maternal and paternal homologues [76]. However, since *E. cuniculi *is not thought to undergo meiosis, this role is unlikely to be important. Budding yeast Hrr25p is also an antagonist of calcineurin signalling by regulating the nuclear localisation and hence activity of the NFAT family transcription factor Crz1p [77]. This is a conserved Ca^2+^-signalling pathway that, amongst other processes, is involved in T-cell activation in mammals, although in *S. cerevisiae *it responds to a variety of environmental stresses that lead to elevated intracellular Ca^2+^, such as high salt and alkaline pH. Further work will be required to determine whether either of these two critical roles are the focus of the *E. cuniculi *CK1 homologues.

### CMGC family

**Figure 4:**
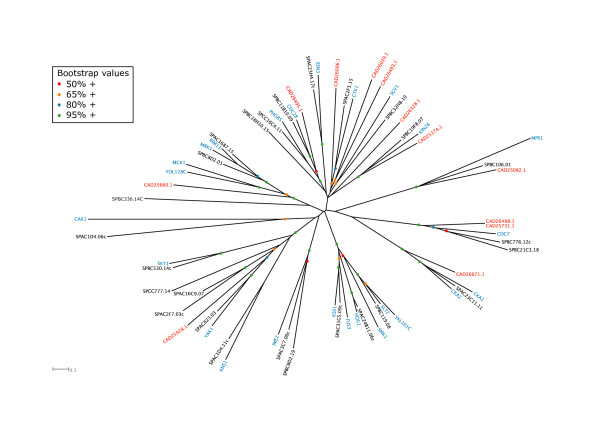
Unrooted tree of the CMGC kinases of *S. cerevisiae *(blue), *S. pombe *(black) and *E. cuniculi *(red).

The CMGCs (cyclin-dependent kinases, mitogen-activated protein kinases, glycogen synthase kinases [GSK3], and CDK-like kinases [CKLs]) are the largest family of kinases in the *E. cuniculi *genome and 8/12 microsporidian CMGCs can be assigned homology to a number of yeast CMGCs that play essential roles (Table 3, Fig. 4, Additional file 4). CAD26498.1 and CAD25731.1 are the microsporidian homologues of budding yeast Cdc7p, which has two paralogues in *S. pombe*, one of which (Hsk1) is also essential. These kinases are DDKs (Dbf4-dependent kinases), so called because they are activated by binding to a regulatory subunit (Dbf4p in *S. cerevisiae*), and they play a fundamental role in the activation of licensed replication origins [78]. The other characterised microsporidian CMGCs are homologues of essential cyclin-dependent kinases such as Cdc28p/Cdc2 (CAD26495.1) and Kin28p/Crk1p (CAD25174.1), the homologue of yeast casein kinase II (CAD26671.1), the homologue of the dual-specificity kinase Yak1p (CAD25928.1), and the homologue of yeast TTK (Mps1p/Mph1: CAD25082.1).

Of the cyclin-dependent kinases, Cdc28p/Cdc2 have clearly established fundamental roles in cell division, while the other yeast CDKs are involved in transcriptional regulation through modulating the phosphorylation state of the RNA pol II C-terminal domain (Ctk1p, Kin28p for example: see [29]). Thus although *E. cuniculi *does not show the full range of CDKs found in the yeasts, it nonetheless has homologues corresponding to both these classes, indicating that the roles of CDKs as cell cycle and RNA pol II regulators are presumably fundamental. Furthermore, at least two of the semi-orphan CMGC kinases (CAD26039.1 and CAD26328.1) may also belong to the CDK group, although we have not classified them as such because they show considerable divergence from any of the conserved fungal CDKs. In common with many protein kinases, several CDKs require activatory phosphorylation on their Tloop threonine, which is carried out in budding yeast by Cak1p [79]. In metazoans, this role is carried out by Cyclin H-Cdk7 rather than by a single subunit Cak1p homologue. Both systems are present in fission yeast, although the Cak1p orthologue (Csk1) appears to be responsible for activating the Cdk7 orthologue (Crk1, also termed Mcs6), which is the direct CDK activator *in vivo *[80]. Since we have found an *E. cuniculi *orthologue of fission yeast Crk1 (CAD25174.1, Table 3), it is therefore likely that this Cdk7-related kinase is responsible for direct phosphorylation of its other CDKs and that there is no Cak1p-related kinase.

Both yeasts have a TTK member, which is known to play roles both in spindle pole duplication and in the spindle checkpoint response that monitors attachment of chromosomes to the mitotic spindle [81]. The spindle pole duplication role is conserved in mammals [82] although apparently not in fission yeast [83], and it is this function that makes *MPS1 *essential in *S. cerevisiae*. Since *E. cuniculi *lacks a Bub1p homologue (see above), another key kinase in the checkpoint pathway, it is likely that the microsporidian TTK (CAD25082.1) is involved primarily in spindle pole duplication.

Yeast Yak1p is a member of the conserved DYRK sub-family that is represented in fission yeast by the as yet uncharacterized Ppk15 (Table 3). Budding yeast Yap1p is involved in glucose signalling [84] and is associated with growth inhibition, functioning in an antagonistic manner either downstream of or in parallel with the PKA pathway [85,86]. The presence of a DYRK member in *E. cuniculi *is therefore consistent with the presence of a PKA orthologue and suggests that the functional relationship between PKA and DYRK has been conserved in the microsporidian.

Casein kinase II is a multifunctional enzyme with roles in processes as diverse as cell cycle progression, cell polarity and ion homeostasis [87,88]. The presence of an *E. cuniculi *orthologue (CAD26671.1) underlines the fundamental importance of this group of kinases and is consistent with the identification of a Casein Kinase II regulatory subunit (CAD25839.1).

Comparing the two model yeasts, most members of the CMGC family show orthologous relationships and there are few apparently lineage-specific 'semiorphans' (Table 3). Members of this family that are found in the yeasts but not the microsporidian include the MAP kinases (involved in stress-activated signal transduction pathways and mating: [89-92]), members of the RCK sub-group (involved in yeast meiotic regulation: [93,94]) and GSK3, which in budding yeast is involved in meiotic induction and in heat stress tolerance [95].

### STE family

**Figure 5:**
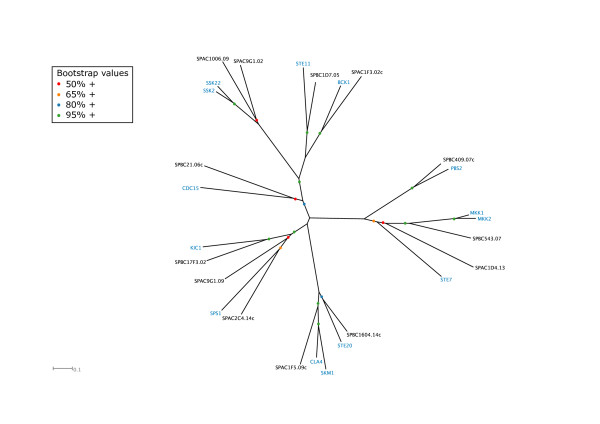
Unrooted tree of the STE kinases of *S. cerevisiae *(blue), *S. pombe *(black) and *E. cuniculi *(red).

All the STE kinases (a family including many kinases functioning in MAP kinase cascades) of *S. cerevisiae *and *S. pombe *were found to share homology relationships (Table 3, Fig. 5, Additional file 5), but no kinases of the STE family were found in *E. cuniculi*. The presence of putative MAPKKK-MAPKK-MAPK modules in the kinomes of the three Trypanosomatid species [19] suggests that these are likely to have been lost in a number of reduced kinomes such as those of *P. falciparum *[17], other Apicomplexa (Miranda-Saavedra, D. *et al*, submitted) and *E. cuniculi*. However, a number of key STE family members function in pathways distinct from MAP kinase pathways in the yeasts, for example Cdc15p/Cdc7 (discussed above), which forms part of the MEN/SIN late mitotic network. Some *STE20 *family members, such as budding yeast Ste20p itself, function upstream of MAP kinase pathways [89]. However, not all of their roles are mediated in this way and so it seems that these other roles are not required in *E. cuniculi*. Several of the STE kinases are Rho GTPase-activated kinases (for example budding yeast Cla4p, Kic1p and Ste20p), characterised by a PB domain that binds the p21 GTPase and sometimes a PH domain upstream of this [96].

### Other protein kinases (OPKs)

**Figure 6:**
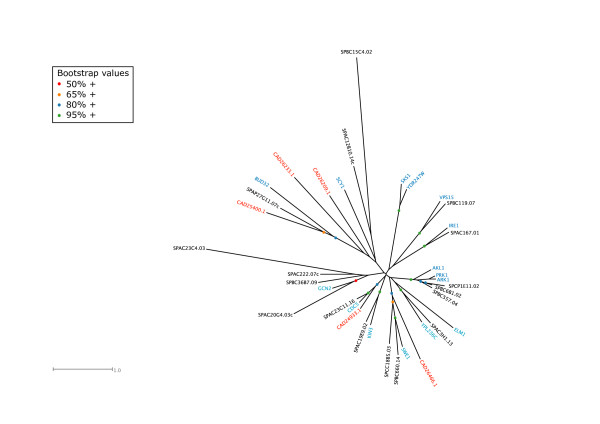
Unrooted tree of the 'Other' kinases of *S. cerevisiae *(blue), *S. pombe *(black) and *E. cuniculi *(red).

This group is constituted by ePKs that cannot be classified confidently into any of the main ePK families. The multi-level HMM library has been used to classify some of the 'Other' kinases of *S. cerevisiae *into the main ePK families by comparison with syntenic homologous genes of the related fungus *Ashbya gossypii *[12]. However, some of the 'Other' kinases of *S. cerevisiae *are likely to constitute yeast-specific families in their own right, and whose identity will emerge upon examination of a larger number of fungal kinomes. Of the 5 microsporidian kinases included in the 'Other' group, only 3 could be mapped to homologous proteins in *S. cerevisiae *and *S. pombe *(Table 3, Fig. 6, Additional file 6). CAD25400.1 is the homologue of Bud32, which we have also now shown to be present in fission yeast. Although named for its apparent role in *S. cerevisiae *bud site selection [97], more recent studies have identified Bud32p kinase as a component of a conserved protein complex with important roles in transcription and telomere maintenance [98,99], and it is likely that these roles explain its presence in *E. cuniculi*.

CAD24933.1 is the orthologue of the essential *polo *kinase (Cdc5p and Plo1 in *S. cerevisiae *and *S. pombe*, respectively), a conserved cell cycle regulatory kinase with many important roles in centrosome and spindle function, sister chromatid cohesion, kinetochore function and mitotic exit [100]. A key feature of Polo kinases is the presence of tandem Polo Box sequences (Pfam PF00659) in the C-terminal nonkinase domain, which like 14-3-3 proteins are a phosphopeptide binding domain that target the kinase to substrates phosphorylated by other kinases [100]. Although the *E. cuniculi *kinase lacks these characteristic C-terminal Polo boxes in a Pfam domain search, manual inspection of the C-terminal region provides evidence for two degenerate Polo box sequences, confirming the identity of this kinase as a Polo homologue.

The third readily-assignable *E. cuniculi *kinase in the 'Other' group is an orthologue of budding yeast Swe1p and fission yeast Wee1 and Mik1 (Table 3). These kinases are negative regulators of the Cdc28p/Cdc2 CDK kinases that regulate the time of entry into mitosis [101-104]. This is an essential and critical role in fission yeast, where loss of both paralogous kinases causes catastrophic premature mitotic entry, whereas in budding yeast the effects of Swe1p are more subtle and cells can manage without it, at least under normal circumstances.

Protein kinases in the 'Other' group that are shared by the two model yeasts but that are not conserved in the microsporidian include Gcn2p (involved in amino acid sensing: [105]), Ire1p (required for the unfolded protein response: [106]), the Ark1p-related kinases required for regulating cortical actin function and endocytosis [107] and Vps15p (required for targeting proteins to the vacuole: [108]), and their fission yeast orthologues.

### Atypical protein kinases

Only aPKs of the PIKK (phosphatidyl inositol 3' kinase-related kinases) and RIO ('*ri*ght *o*pen reading frame') families were identified in the *E. cuniculi *genome, and putative homology could be assigned in all three cases. CAD25142.1 and CAD25955.1 are related to budding yeast Tel1p and are likely to be involved in telomere maintenance and the DNA damage checkpoint response as discussed above. The TOR group of PIKK members are involved in nutrient sensing pathways and are not represented in the microsporidian [109]. A homologue of Tra1p, apparently conserved between the two yeast species, was also not evident despite its essential role as a core component of the SAGA and NuA4 histone acetyl transferase complexes in budding yeast that are important for transcriptional activation, particularly involving acidic activators [110,111]. It is not clear how *E. cuniculi *can dispense with such a function, although many (but not all) of the components of the yeast SAGA and NuA4 complexes are not essential for viability (see [25]). The Rio kinases are required for 20S prerRNA processing [112,113], a role which is apparently conserved in *E. cuniculi*. Finally, *E. cuniculi *lacks a pyruvate dehydrogenase (PDH) kinase, an enzyme that downregulates PDH activity by phosphorylation of the E1 subunit [114]. The status of PDH in *E. cuniculi *is currently somewhat equivocal, since the microsporidian has two E1 subunit homologues but no evident E2 or E3 component [115], and the E2 component is critical for regulating PDH kinase activity [116]. Thus without a complete PDH complex there is probably no need for a PDH kinase. With the exception of one of its two PDH kinases, budding yeast aPKs all show clear orthologous relationships to their fission yeast counterparts.

### Protein kinase accessory domains

Only 2 ePKs belonging to the AGC family were found to contain readily identifiable domains in addition to the kinase catalytic domain. These are the protein kinase Cterminal domain (PF00433) and the protein kinase C phorbol ester/diacylglycerolbinding domain (PF00130). The microsporidian PKA (CAD25584.1) presents the domain architecture NH_3 _^+^-kinase-PF00433-CO_2 _^-^. The protein kinase C-terminal domain is found in a variety of proteins with different functions and dependencies, and so *per se *it is not useful for assigning putative function. The AGC kinase CAD25005.1 presents the domain organisation NH_3 _^+^kinasePF00433PF00130-CO_2 _^-^.

### Protein kinase-regulating proteins

Only two cyclins were found in the *E. cuniculi *genome (CAD26331.1 [Q8SRF2] and CAD27077.1 [Q8STR3]). One instance of the regulatory subunit of Casein Kinase II was also found (CAD25839.1 [Q8SR24]), plus two regulatory subunits of PKA (CAD24891.1 and CAD25013.1).

## Conclusion

The 2.9 Mb genome of the microsporidian *E. cuniculi *is the smallest known for any eukaryote. A massive gene loss in the fungal clade, with additional elimination in *E. cuniculi*, has been inferred from the reconstruction of parsimonious evolutionary scenarios using either a subset of KOGs or "eukaryotic orthologous groups" [117] or the complete collection [118]. The common ancestor of *E. cuniculi *and two yeast species is predicted to contain 3,048 KOGs, and the branch leading to the microsporidian would be characterised by 586 gene gains and up to 1,969 gene losses. The *E. cuniculi *proteome appears as a package of compact proteins containing a significant proportion of orthologues with simplified domain organisation or with a high frequency of intragenic deletions [118]. From the analysis of the protein size distributions derived from sequenced genomes, it can be suggested that the lengthening of proteins in eukaryotes (non-parasitic species) allows for more complex regulation networks. Thus, protein shortening in *E. cuniculi *may reflect reduced protein-protein interactions as a result of various gene losses linked to the intracellular parasitic nature [2]. The kinome of *E. cuniculi*, consisting of only 32 protein kinases (29 ePKs and 3 aPKs), is a good illustration of this hypothesis.

The microsporidian kinome is approximately one fourth the size of the kinomes of *S. cerevisiae *(115 ePKs and 9 aPKs) and *S. pombe *(109 ePKs and 8 aPKs). The *E. cuniculi *kinome has underscored the importance of a number of protein kinases that are involved in essential cellular processes and likely to be essential to all eukaryotes. Therefore, the microsporidian presents an opportunity for evaluating the basic aspects of the most fundamental cellular mechanisms as mediated by protein kinases. The *E. cuniculi *kinome includes what might be considered as a core set of protein kinases required for performing the cell division cycle: a Cdc28p/Cdc2 cyclin-dependent kinases to regulate progression through different cell cycle stages, its negative regulator (Swe1p/Wee1 orthologue), a DDK to trigger initiation of DNA replication, a *polo *kinase an Aurora kinase to orchestrate various aspects of cell division, a TTK for spindle pole duplication and homologues of Te1lp and Chk1p for regulation in response to DNA damage and/or stalled replication forks. A second CDK might also function as a CDK-activating kinase, and the Bud32p orthologue may be needed for telomere maintenance. Kinases involved specifically and fundamentally in cell cycle regulation may therefore represent ~30% of the *E. cuniculi *kinome, and orthologues of all the critical activities appear to be present with the exception of those that form part of the fungal MEN/SIN pathways.

In contrast, *E. cuniculi *appears to lack almost all of the protein kinases involved in stress responses, ion homeostasis and nutrient signalling. Although it has orthologues of PKA and DYRK, there is a complete lack of MAP kinase pathways and many other kinases involved in these signalling routes. Most notable by their absence are TOR and AMPK, and *E. cuniculi *may be the first eukaryote in which neither of these conserved functions is found. These striking differences with other eukaryotes presumably relate to its specialised, intracellular, lifestyle as an obligate parasite. Within its parasitophorous vacuole, it can rely on the host cell to provide nutrients, ATP and an osmotically stabilized environment that must be relatively unchanging compared to that of the free-living yeasts. Since *E. cuniculi *is not thought to undergo meiosis, the absence of orthologues to the yeast meiotic kinases is also hardly surprising.

9/32 (28.1%) of the microsporidian kinases are considered as 'semi-orphan' in our analysis, not showing clear orthologous relationships to any of the yeast kinases. This emphasizes the rapid evolution of some genes in *E. cuniculi *and these kinases may be involved in functions related to the parasitic lifestyle of the organism, for example the decision to initiate spore development (which is likely not to be nutrient-regulated as in yeasts), or regulation of the polar tube that is used for cell invasion [115]. In contrast, perhaps the most striking aspect of the comparison between the kinomes of *S. cerevisiae *and *S. pombe *is the extent to which orthologous relationships are clear: only ~15% of kinases in each yeast could not be assigned such relationships despite at least 800 million years of divergent evolution [119]. We hope that the comparison between the kinomes of *S. cerevisiae *and *S. pombe *will stimulate research into many of the as yet uncharacterised fission yeast kinases.

## Methods

### Identification and classification of *E. cuniculi *protein kinases

The set of predicted peptides of *E. cuniculi *was downloaded via the Sequence Retrieval System [120]. The set of 5003 predicted peptides of *S. pombe *was downloaded from the *S. pombe *Genome Project [121]. The retrieval of protein kinases and their automatic classification into protein kinase families was done by scanning the predicted peptides with a multi-level hidden Markov model library of the protein kinase superfamily run under HMMER v.2.1.1 [122,123]). This HMM library has been developed to identify and sub-classify protein kinase catalytic domains into one of the accepted conventional (ePK) and atypical (aPK) protein kinase families. The library has been shown to have a misclassification rate of zero on the family level and for the annotated kinomes of *H. sapiens, M. musculus, C. elegans, S. cerevisiae, P. falciparum*, and *D. discoideum *[12].

### Phylogenetic analysis

Following the generation of multiple alignments for the kinase catalytic domains of each kinase family of *E. cuniculi, S. cerevisiae*, and *S. pombe*, these were inspected and curated for large insertions and misaligned regions. The final curated alignments were of a length of no less than 220 amino acids, in agreement with the size of the kinase catalytic domain (~250 amino acids). SplitsTree [124] was used to generate the phylogenetic trees with the JTT matrix and the Neighbour-Joining algorithm. The bootstrap values reported here are based on 1000 replicates. Family-specific dendrograms derived from complete linkage clustering of kinase catalytic domains were eventually built to assist in the phylogenetic analysis.

### Identification of accessory domains, transmembrane helices, and protein kinase-regulating proteins

The full-length protein kinases of *E. cuniculi *were scanned with a local installation of InterProScan [125,126] run with default parameters. Transmembrane helices were predicted with TMHMM 2.0 [127,128]. Protein kinase-regulating subunits were identified with the Pfam HMMs (Pfam_fs versions) PF01214 (regulatory subunit of Casein Kinase II), PF00134 and PF02984 (cyclins), PF02197 (PKA regulatory subunit) and PF03941 (Sli15p).

## Abbreviations

ePK, conventional protein kinase; aPK, atypical protein kinase; CDK, cyclin-dependent protein kinase; CDDK, Dbf4-dependent protein kinase; DYRK, dual specificity tyrosine phosphorylated and regulated kinase; HMM, hidden Markov model; KOGs, eukaryotic orthologous groups; MEN, mitotic exit network; PKA, cyclic AMP-dependent protein kinase; SIN, septation initiation network.

## Authors' contributions

CD and JCP carried out preliminary E. cuniculi database searches and phylogenetic analyses that initiated this study. DMS carried out the analysis presented here and wrote the larger part of the manuscript. CD, GJB, MJRS and CPV contributed to the writing of this manuscript. All authors read and approved the final manuscript.

## Supplementary Material

Additional file 1 Multiple sequence alignment of AGC family kinase catalytic domains from *S. cerevisiae, S. pombe *and *E. cuniculi *prior to curation for phylogenetic analysis. Click here for file

Additional file 2 Multiple sequence alignment of CAMK family kinase catalytic domains from *S. cerevisiae, S. pombe *and *E. cuniculi *prior to curation for phylogenetic analysis. Click here for file

Additional file 3 Multiple sequence alignment of CK1 family kinase catalytic domains from *S. cerevisiae, S. pombe *and *E. cuniculi *prior to curation for phylogenetic analysis. Click here for file

Additional file 4 Multiple sequence alignment of CMGC family kinase catalytic domains from *S. cerevisiae, S. pombe *and *E. cuniculi *prior to curation for phylogenetic analysis. Click here for file

Additional file 5 Multiple sequence alignment of STE family kinase catalytic domains from *S. cerevisiae, S. pombe *and *E. cuniculi *prior to curation for phylogenetic analysis. Click here for file

Additional file 6 Multiple sequence alignment of Other family kinase catalytic domains from *S. cerevisiae, S. pombe *and *E. cuniculi *prior to curation for phylogenetic analysis. Click here for file
